# Effect of phenylacetamide isolated from *lepidium apetalum* on myocardial injury in spontaneously hypertensive rats and its possible mechanism

**DOI:** 10.1080/13880209.2020.1778043

**Published:** 2020-07-06

**Authors:** Qi Zhang, Peipei Yuan, Meng Li, Yang Fu, Ying Hou, Yaping Sun, Liyuan Gao, Yaxin Wei, Weisheng Feng, Xiaoke Zheng

**Affiliations:** a College of Pharmacy, Henan University of Chinese Medicine, Zhengzhou, China; b Co-construction Collaborative Innovation Center for Chinese Medicine and Respiratory Diseases by Henan & Education Ministry of P.R. China, Zhengzhou, China

**Keywords:** H9c2, anti-myocardial injury, antihypertensive

## Abstract

**Context:**

In the antihypertensive study of phenylacetamide (PA) on spontaneously hypertensive rats (SHR), it was occasionally found that PA prevents myocardial injury.

**Objective:**

Clarify the protective mechanism of PA on myocardial injury in SHR rats.

**Materials and methods:**

*In vivo*, SHR rats were treated with or without PA (15, 30, 45 mg/kg) for 3 weeks (12 per group). *In vitro*, H9c2 cells were treated with PA (1, 5, 10 μM) for 24 h, and then stimulated with H_2_O_2_ (300 μM) for 4 h. Molecular mechanisms were explored through cardiac pathology, cardiac function and biochemical markers.

**Results:**

*In vivo*, PA (15, 30, 45 mg/kg) reduced CVF from 14.8 ± 1.62 to 9.94 ± 1.56, 8.6 ± 1.33, 8.14 ± 1.45%; increased the LVEF relative level from 0.8 ± 0.06 to 0.83 ± 0.04, 0.86 ± 0.05, 0.9 ± 0.04. All three doses can improve the cardiac pathological structure and function (LVEDD, LVESD, LVFS, heart index, NT-proBNP, CKMB, SBP); however, 45 mg/kg works best. But different doses show different molecular mechanisms. PA (15 mg/kg) improves RAAS system (REN, ACE), inflammation (ET-1, IL-1β) and MAPK pathway (p-ERK/ERK, p-JNK/JNK) better. PA (45 mg/kg) improves oxidative stress (SOD, NOX1) and TGF-β pathway (Smad3) better. *In vitro*, PA improved cell viability, oxidative stress (SOD, NOX1) and Smad3 protein expression.

**Discussion and conclusions:** PA regulates different mechanisms at different concentrations to improve myocardial injury, and high dose is the best. This experiment provides a theoretical basis for the development of new clinical drugs for cardiovascular disease.

## Introduction

*Lepidium apetalum* Willd. (Brassicaceae) dry mature seed, recorded in ‘Shennong’s Classic of Materia Medica’, is a medicinal material mainly used in edoema syndrome (National Pharmacopoeia Commission 2015). At present, there are few reports about the pharmacodynamics of *Lepidium apetalum*. In this study, the main active component phenylacetamide, was isolated from the seeds of *Lepidium apetalum*. During a study of the antihypertensive effect of phenylacetamide, it was found that phenylacetamide could improve myocardial injury in rats with spontaneous hypertension.

Hypertension is the cause of many cardiovascular diseases affecting one-third of the global population (Harrison 2014). Long-term hypertension can lead to decreased left ventricular systolic function (Cezar et al. 2013). As common markers to measure the degree of myocardial injury, creatine kinase MB isoenzyme (CKMB) and *n*-terminal pro-brain natriuretic peptide (NT-proBNP) are widely used in predicting the prognosis of heart failure induced by various causes (Hedstrom et al. 2007; Shon et al. 2017; Stämpfli et al. 2017). In addition, the local renin-angiotensin (RAS) system in the heart plays a particularly important role in myocardial damage caused by hypertension (Sandhya et al. 2005; Tsai et al. 2008). This is reflected in some patients with hypertension-induced myocardial ischaemia, in which the RAS system is not activated, but the use of angiotensin-converting enzyme inhibitors can still show a good therapeutic effect (Hollenberg 1999). Several studies have confirmed that oxidative stress and inflammatory reactions exist in patients with hypertension and may be involved in the pathogenesis of the disease (Zou et al. 2016).

Mitogen-activated protein kinase (MAPK) is a highly evolutionarily conserved serine/threonine kinase (Mazzon et al. 2012). In patients with hypertension, MAPK is activated by local RAS activation, oxidative stress, inflammation, and other extracellular stimuli, which is the key signal transduction system involved in many important cellular physiological or pathological processes (Esposito et al. 2010; Lawrence et al. 2008). Extracellular regulated protein kinase (ERK1/2), c-Jun N-terminal kinase (JNK) and p38 mitogen-activated protein kinase (p38 MAPK) are three important subfamilies in the MAPK signalling pathway (Liang and Yang 2019). The transforming growth factor β (TGF-β) superfamily is involved in the regulation of differentiation, senescence, immune response, callus, apoptosis and other important life processes (Whitman 1998). Smad3 is the key protein for its downstream involvement in myocardial fibrosis (Kashyap et al. 2017). Interestingly, some scholars have found that the use of p38 MAPK inhibitors can significantly improve right ventricular function and improve myocardial fibrosis (Kojonazarov et al. 2017). The two signalling pathways of MAPK and TGF-β are in mutual ‘dialogue’ to participate in the myocardial injury caused by hypertension (Kacimi and Gerdes 2003).

Therefore, SHR and H_2_O_2_-induced H9c2 injury model were used in this study to clarify the effect of phenylacetamide on cardiac function in SHR by echocardiography.

## Materials and methods

### Animals and cells

Twelve eight-week-old male Wistar-Kyoto Rats (WKY) and 60 eight-week old spontaneously hypertensive rats (SHR) all with body weights of 180–220 g were purchased and maintained under standard laboratory conditions (21 ± 2 °C; 60–65% humidity) at 14/10 h light and dark cycle in a polycarbonate cage. Animal licence numbers were: SCXK (Beijing) 2017-0011, 2017-2001 (purchased from Beijing Vital River Laboratory Animal Technology Co., Ltd). All experiments were performed in accordance with the experimental animal management method issued by the Ministry of Science and Technology of China and approved by the Animal Ethics Committee of Henan University of Chinese Medicine. In addition, all the animals were handled based on the guidelines formulated by the National Institute of Health (NIH) containing Guide for the care and handling of laboratory animals.

The rat cardiomyocyte cell line H9c2 was purchased from the Shanghai Cell Bank of the Chinese Academy of Sciences.

### Drug

The seeds of the *Lepidium apetalum* were picked from Nanyang, Henan Province in 2015, and were identified as the dry mature seeds of the plant Professor Chengming Dong of the College of Pharmacy, Henan University of Chinese Medicine. The voucher specimen is number 2015-TLZ.

**Figure 1.**
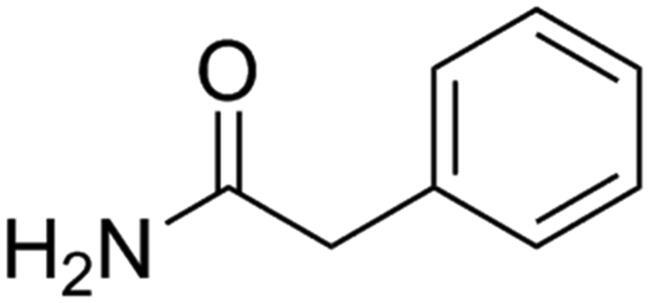
Structure of phenylacetamide. The extraction purity is higher than 98%.

In the early stage of the research group (Meng et al. 2016), the seeds were heated at 240 °C and continuously stirred for 5.5 min to obtain processed product. The processed product (8 kg) was extracted three times with 80 L water for 1.5 h each time. The aqueous portions were combined and then concentrated under reduced pressure to obtain 270 g of extract. The extract was precipitated for 2 h with 20 L of 80% ethanol. The precipitate was discarded, and the supernatant was centrifuged and concentrated to 1 L. The supernatant was separated on the Dianion HP-20 column and eluted with 20 L of water, 60 L of 20% ethanol and 60 L of 40% ethanol, and the flow rate was 20 L/h. The eluting fraction of 40% ethanol was collected, dissolved in water, filtered by centrifugation, concentrated to 35 mL separated by Toyopearl HW-40 column and eluted with 3 L of water and 10% methanol aqueous solution at a flow rate of 8 mL/min. The elution part of 10% methanol aqueous solution was collected, dissolved with methanol for 24 h, colourless crystallisation was precipitated, the upper layer solution was sucked out, and colourless crystal was dried, dissolved again with methanol for 24 h, and so repeated 5 times. The colourless crystal was phenylacetamide (4.3 g), and the purity is more than 98% by NMR. Compound number is TLZ-4-1 (Figure 1).

### *In vivo* experiment

#### Grouping and doses

WKY was used as the normal control (WKY) group and according to the principle of uniform body weight and blood pressure, SHR was divided into five groups: model (SHR) group, hydrochlorothiazide (SHR + HCTZ) (Jiangsu Vanguard Pharmaceutical, Chain) group (17 mg/kg), low (SHR + PA-L), middle (SHR + PA-M) and high (SHR + PA-H) doses of phenylacetamide group (15, 30, 45 mg/kg) were given orally for three weeks.

### Measurement of systolic blood pressure

Systolic blood pressure (SBP) of rats was measured by non-invasive tail artery blood pressure instrument every week (Techman, China).

### Echocardiography

Cardiac echocardiographic parameters, including left ventricular end diastolic diameter (LVEDD), left ventricular end systolic diameter (LVESD), left ventricular ejection fraction (LVEF) and left ventricular short axis shortening rate (LVFS) were measured by small animal echocardiography. The data of each group were measured three times in parallel, and the average values were analysed statistically.

### Detection of antioxidant enzyme SOD

To evaluate the effect of phenylacetamide on oxidative stress injury of SHR, the activity of SOD was measured by the WST-1 method (Nanjing Jiancheng Bioengineering Institute, China). WST-1 can react with superoxide anion catalysed by xanthine oxidase to produce water soluble methanogen dyes. This reaction step can be inhibited by SOD. The enzyme activity of SOD can be calculated by colorimetric analysis of WST-1 products.

### Enzyme-linked immunosorbent assay (ELISA)

Myocardial injury marker NT-proBNP and CKMB, cardiac local RAS system-related indicators REN and ACE, pro-inflammatory factor markers ET-1 and IL-1β were determined by double antibody sandwich assay in enzyme-linked immunosorbent assay (Elabscience, China). The solid-phase carrier containing the corresponding solid-phase antibody was taken, and the sample or standard material was added to bind to the solid-phase antibody and washed to remove the unbound component. The biotinylated anti-antigen antibody was added to bind specifically with the antigen to be tested on the solid phase carrier, and the unbound components were removed by washing. Horseradish peroxidase (HRP)-labelled avidin was added to form immune complex with biotin. The unbound components were removed by washing, and the substrate 3,3′,5,5′-tetramethylbenzidine (TMB) was added to colour. After the discolouration, the optical density (OD) value was detected at 450 nm by an enzyme labelling instrument (Imark Microplate Reader, USA).

### Western blot

The expression of phosphorylation of ERK1/2, JNK and p38 MAPK protein in the MAPK signalling pathway (Cell Signalling Technology, USA), TGF-β superfamily receptor Smad3 (ABClonal, China) and nicotinamide adenine dinucleotide phosphate (NADPH) oxidase homolog NOX1 (Proteintech, China) were detected by western blot. Myocardial tissue frozen at -80 °C was washed rapidly in normal saline, homogenised in phenylmethylsulfonyl fluoride (PMSF)-containing lysate with homogeniser, incubated on ice for 3 h, centrifuged for 10 min with 12,000 rcf (Eppendorf Centrifuge 5804/5804 R, Germany), the supernatant was extracted and the protein was quantified by BCA method. The total protein (40 μg) was separated by sodium dodecyl sulphate-polyacrylamide gel electrophoresis (SDS-PAGE) according to the molecular weight and transferred onto a polyvinylidene fluoride (PVDF) membrane using the semi-dry method. After the membrane-containing protein was immersed in 5% skimmed milk powder to seal the blank site, the first antibody specific to the antigen to be tested was added to incubate for the night, the unbound antibody was removed by washing, the second antibody with fluorescent group was added and the unbound second antibody was removed by washing. An Odyssey dual-colour infra-red fluorescence imaging system (Odyssey, USA) was used to scan and quantify the data.

### Routine staining and immunohistochemistry of myocardial pathology

Production of pathological slices: After collecting the material, the mung bean size tissue at the apex of the rat hearts were quickly cut and fixed in 3.7% formaldehyde for 24 h, dehydrated with ethanol gradient, embedded with xylene and paraffin, and cut into 4 μm thick slices. Hematoxylin-eosin (HE) and Masson staining: Xylene dewaxing, ethanol gradient rehydration, HE and Masson conventional staining, ethanol gradient elution, xylene transparent, neutral gum mount, microscopic examination under an optical microscope observed myocardial morphology changes. Immunohistochemistry: Xylene dewaxing, ethanol gradient rehydration, antigen repair, 10% normal rabbit serum blocked. Add NOX1 protein primary antibody dilution and incubate at 4 °C overnight. Add the corresponding secondary antibody to incubate, DAPI counterstains the cell nucleus, observes and collects the picture under the inverted microscope after mounting. For the results of Masson staining and NOX1 immunohistochemistry, 5 pictures were randomly taken from each group and analysed with Image-Pro Plus 6.0 software.

### *In vitro* experiments

#### H9c2 cell culture

H9c2 were inoculated into Dulbecco’s modified eagle’s medium (DMEM, Gibco, USA) containing 10% foetal bovine serum (Gibco, USA) under the conditions of 5% CO_2_, 37 °C, and saturated humidity (Shanghai STIK, China). When the cells were grown to 80–90%, they were digested with trypsin (0.25%) containing 0.02% ethylenediaminetetraacetic acid (EDTA) at a ratio of 1:3 or 1:4.

### Thiazolyl blue tetrazolium bromide (MTT) assay for cell viability

H9c2 cells were inoculated into a 96-well culture plate with a density of 5 × 10^4^ per mL. After culturing for 24 h, the cells were divided into 6 groups: the normal (NC) group and the model (M) group added only 10% foetal bovine serum containing DMEM. After phenylacetamide low (H_2_O_2_+PA-L), medium (H_2_O_2_+PA-M), high (H_2_O_2_+PA-H) dose groups were added with 1, 5, 10 mM phenylacetamide for 24 h, respectively, then 300 μM of H_2_O_2_ was added to the M group, and 300 μM of H_2_O_2_ and 1, 5, 10 μM phenylacetamide were added to the low, medium, and high dose groups for 4 h. MTT (5 mg/mL) (Biosharp, China) was added to each hole for 4 h, the supernatant was discarded, and 150 μL of dimethyl sulphoxide (DMSO) was added to each hole to dissolve formazan. OD value at 490 nm was detected by enzyme-labelling instrument.

### Detection of oxidative stress markers

To evaluate the effect of phenylacetamide on oxidative stress induced by H_2_O_2_ in H9c2 cells, the activity of SOD was detected by WST-1 assay.

#### In-Cell Western assay

The expression of NOX1 and Smad3 protein were detected by In-Cell Western assay. H9c2 cells were inoculated evenly in 96-well plate. The treatment method was the same as above operation. Then, the medium abandoned and the cells solidified. The cells were incubated with 3.7% formaldehyde for 20 min at room temperature, and then the cell membrane was permeated by 0.1% triton. After 10 min, it was incubated at room temperature with methanol, and the excess methanol was removed by phosphate-buffered saline (PBS) washing. After curing, 5% skimmed milk powder (without Tween 20) was incubated for 1 h to block the non-specific sites. After blocking, 5% skimmed milk powder (excluding 0.1% Tween 20) diluted specific antibody (primary antibody) was added. After incubation at 4 °C for 16 h, the unbound antibody was removed by washing. 5% Skimmed milk powder (including 0.1% Tween 20) was added, diluted with fluorescent labelled secondary antibody, incubated for 1 h and washed to remove the unbound second antibody, absorb the fluid in the pore and keep the cells moist. Data was scanned and quantified in the Odyssey dual-colour infra-red fluorescence imaging system.

### Detection of reactive oxygen species (ROS) by flow cytometry

H9c2 cells were seeded in a 6-well plate. After the cells grew to 70-80%, they were treated in groups in the same way as above. After washing the cells with PBS, DCFH-DA probe diluted in DMEM (final concentration 10 μmol/L) was added, and cells were incubated at 37 °C for 20 min in the dark and washed three times with PBS to remove unloaded probes. After collecting cells by trypsin and suspending them in PBS, the ROS levels of the cells were quickly detected by BD FACS Aria III flow cytometer (BD, USA).

### Statistical methods

The data were analysed by SPSS 18.0 software and compared with each other by one-way analysis of variance (one-way ANOVA) with Tukey’s Honestly Significant Difference (HSD) for *post hoc* analysis of the experiments. Experimental data is expressed as mean ± SD. *p* < 0.05 indicates that the difference is significant, and *p* < 0.01 indicates that the difference is extremely significant.

## Results

### Effect of phenylacetamide on pathological structure and collagen volume fraction (CVF) of hearts in SHR rats

**Figure 2.**
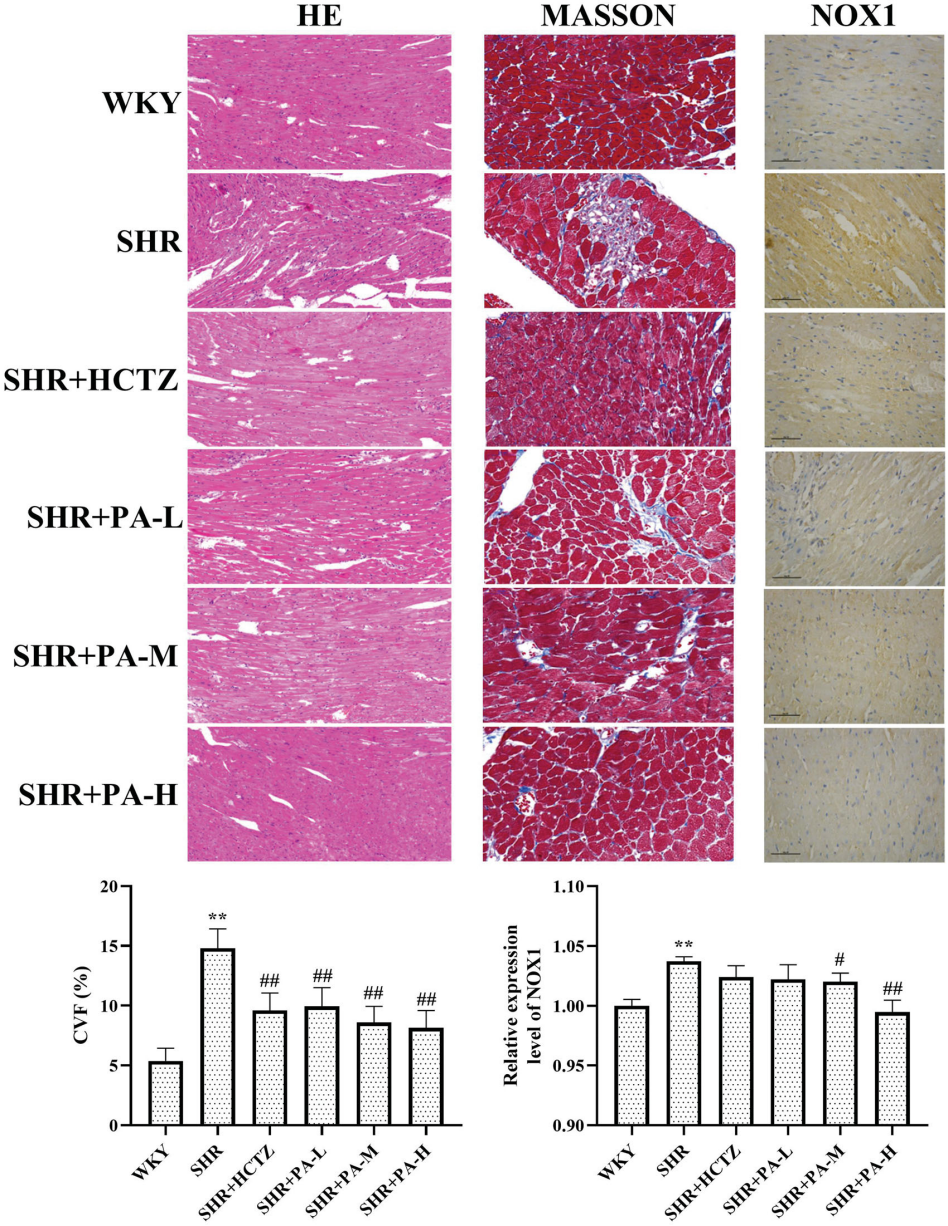
Effects of phenylacetamide on cardiac pathological structure, CVF and NOX1 protein expression in SHR rats. The value of the WKY group of NOX1 was set at 1, and the relative value was presented as fold induction to that of the WKY group. Values are expressed as the mean ± SD of 5 samples. *p < 0.05, **p < 0.01 vs. WKY group; #*p* < 0.05, ##*p* < 0.01 vs. SHR group.

The results showed that in the WKY group, myocardial fibres were neatly arranged, uniform in size, and evenly distributed. The horizontal stripes are clear, the gap is normal, the nucleus is normal, and the collagen fibre is deposited less. In the SHR group, the cardiomyocytes were thickened and enlarged, and the arrangement was disordered. Striae rupture, interstitial edoema, cell nuclei increased, CVF increased obviously (*p* < 0.01). After phenylacetamide treatment, myocardial fibres are arranged relatively neatly. The stripes are clear, the gap is normal, and the nucleus is normal. The CVF is reduced (*p* < 0.01), and the high-dose improvement effect is the best (Figure 2).

### Effects of phenylacetamide on left ventricular systolic function, heart index, serum NT-proBNP and CKMB in SHR rats

**Figure 3.**
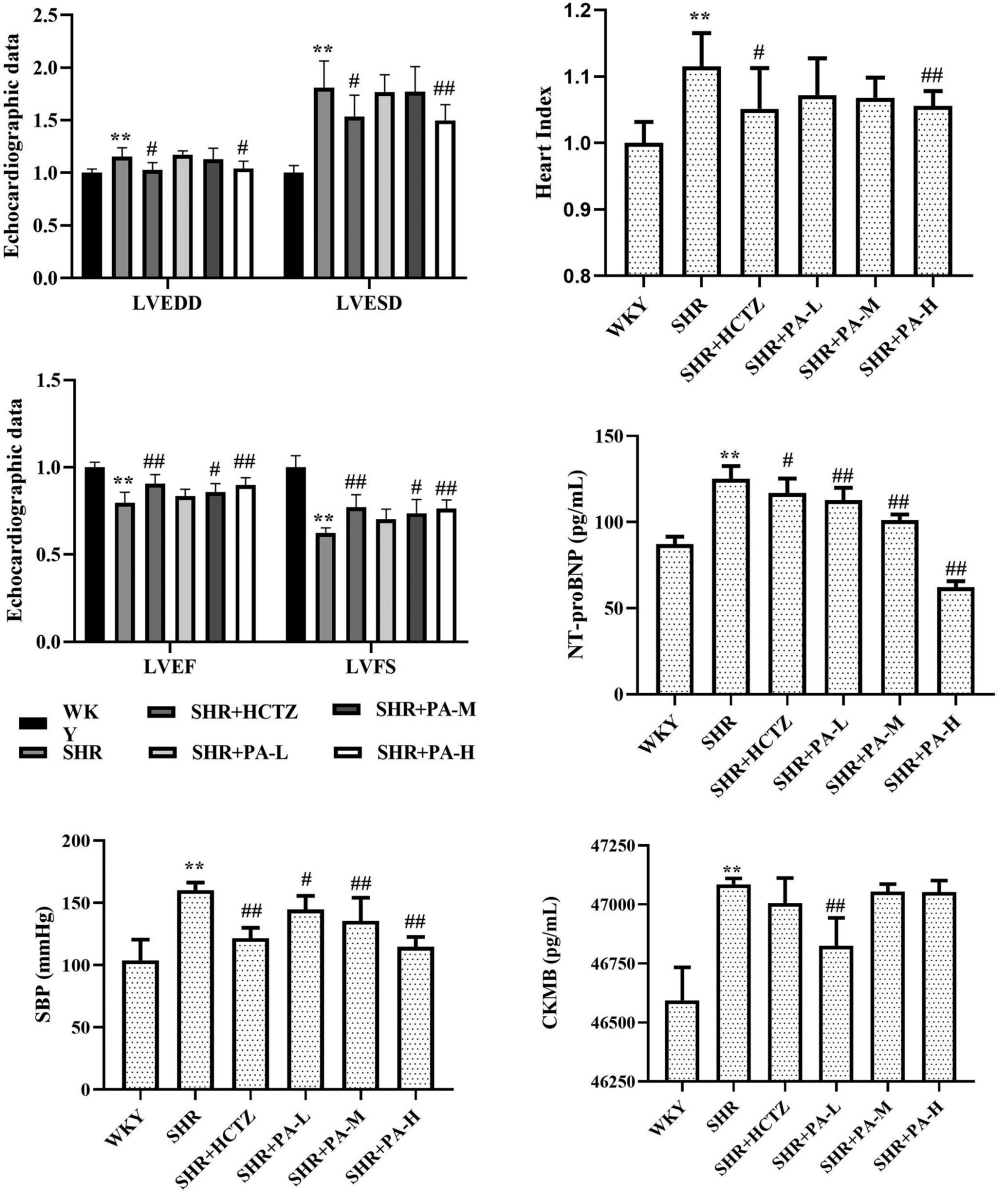
Effects of phenylacetamide on left ventricular systolic function, heart index, NT-proBNP, CKMB, and SBP level in SHR rats. The values of the WKY group of heart index, SBP, LVEDD, LVESD, LVEF and LVFS were set at 1, and the relative values were presented as fold induction to that of the WKY group. Values are expressed as the mean ± SD of 6 samples. *p < 0.05, **p < 0.01 vs. WKY group; #*p* < 0.05, ##*p* < 0.01 vs. SHR group.

Compared with the WKY group, the LVEDD, LVESD, heart index, serum NT-proBNP and CKMB levels in the SHR group were significantly increased (*p* < 0.01), and the LVEF, LVFS levels were obviously lower (*p* < 0.01), suggesting a decrease in left ventricular systolic function. Compared with the SHR group, low-dose phenylacetamide can only obviously improve NT-proBNP and CKMB (*p* < 0.05 or *p* < 0.01); middle-dose phenylacetamide can obviously improve LVEF, LVFS and NT-proBNP (*p* < 0.05 or *p* < 0.01); high-dose phenylacetamide can obviously improve all the above indicators except CKMB (*p* < 0.05 or *p* < 0.01). According to the results of CKMB, it is preliminarily speculated that different doses of phenylacetamide have different effects on the improvement of myocardial injury in SHR rats, but it can be confirmed that the myocardial protection effect of high-dose conditions is stronger (Figure 3).

### Effect of phenylacetamide on SBP in SHR rats

The results showed that compared with the WKY group, the level of SBP in the SHR group was higher (*p* < 0.01). After 3 weeks of drug intervention, it was found that phenylacetamide could reduce the level of SBP (*p* < 0.05 or *p* < 0.01) and alleviate the degree of hypertension. Each dose has significant efficacy, among them, the best effect is at high doses (Figure 3).

### Effect of phenylacetamide on the RAS system in the hearts of SHR rats

**Figure 4.**
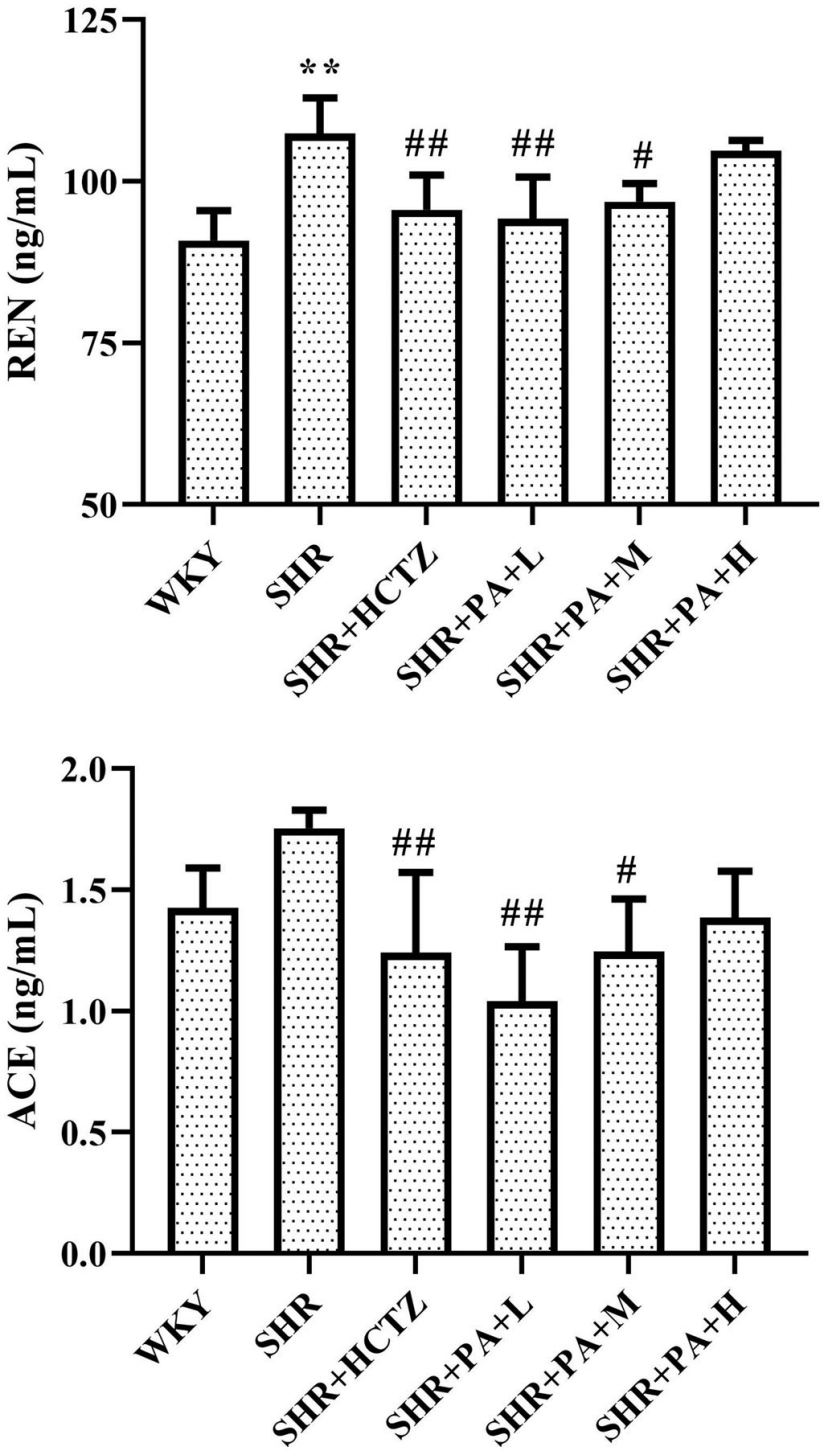
Effects of phenylacetamide on REN and ACE in the hearts of SHR rats. Values are expressed as the mean ± SD of 6 samples. *p < 0.05, **p < 0.01 vs. WKY group; #*p* < 0.05, ##*p* < 0.01 vs. SHR group.

The levels of renin (REN) and angiotensin-converting enzyme (ACE) in myocardial homogenate were measured by ELISA. The results showed that compared with the WKY group, the REN level in the SHR group was obviously increased (*p* < 0.01), and the ACE also showed an increasing trend, indicating that the local RAS system of the SHR rats was activated. Compared with the SHR group, both low- and medium-dose phenylacetamide can inhibit REN and ACE (*p* < 0.05 or *p* < 0.01), thereby inhibiting the excessive activation of the RAAS system. Among them, the low dose works best (Figure 4).

### Effect of phenylacetamide on inflammatory factors in SHR rats

**Figure 5.**
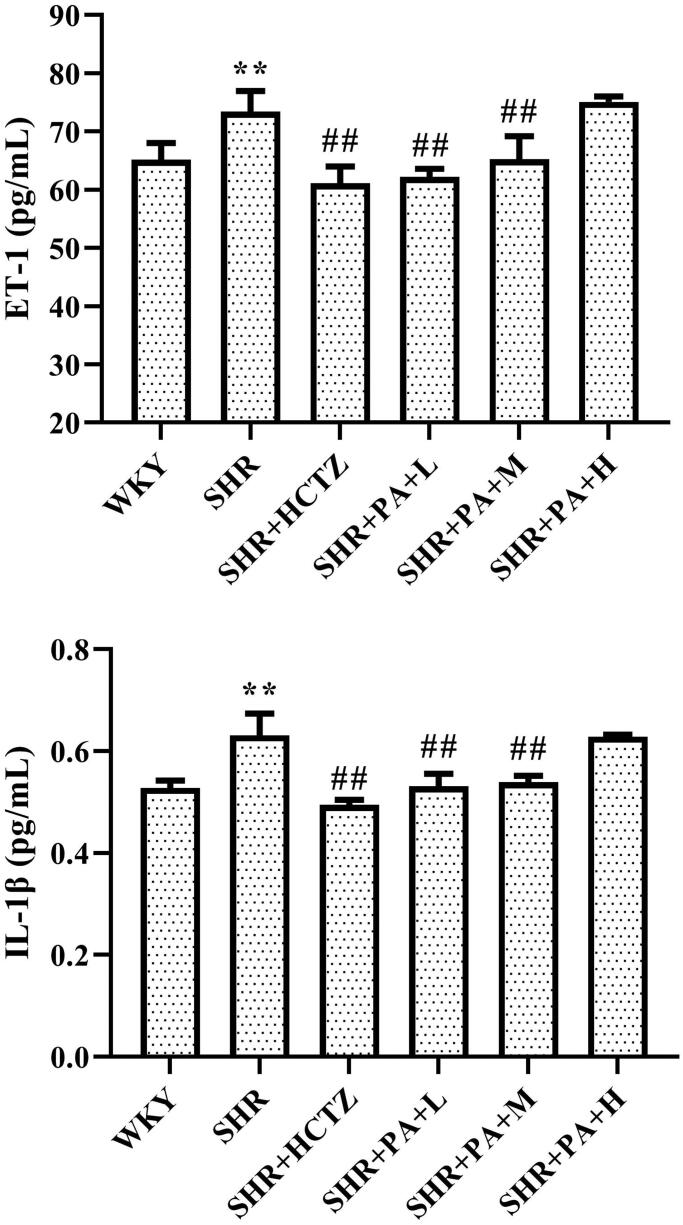
Effects of phenylacetamide on ET-1 and IL-1β in the hearts of SHR rats. Values are expressed as the mean ± SD of 6 samples. *p < 0.05, **p < 0.01 vs. WKY group; #*p* < 0.05, ##*p* < 0.01 vs. SHR group.

The levels of ET-1 and IL-1β in myocardial tissue homogenate were separately detected by ELISA. The results showed that compared with the WKY group, the levels of ET-1 and IL-1β in the SHR group were significantly increased (*p* < 0.01), suggesting that there was an inflammatory reaction in the SHR rats. Compared with the SHR group, both low- and medium-dose phenylacetamide can inhibit ET-1 and IL-1β (*p* < 0.01), thereby inhibiting inflammation. Among them, the low dose works best (Figure 5).

### Effect of phenylacetamide on oxidative stress-related indexes in SHR rats

**Figure 6.**
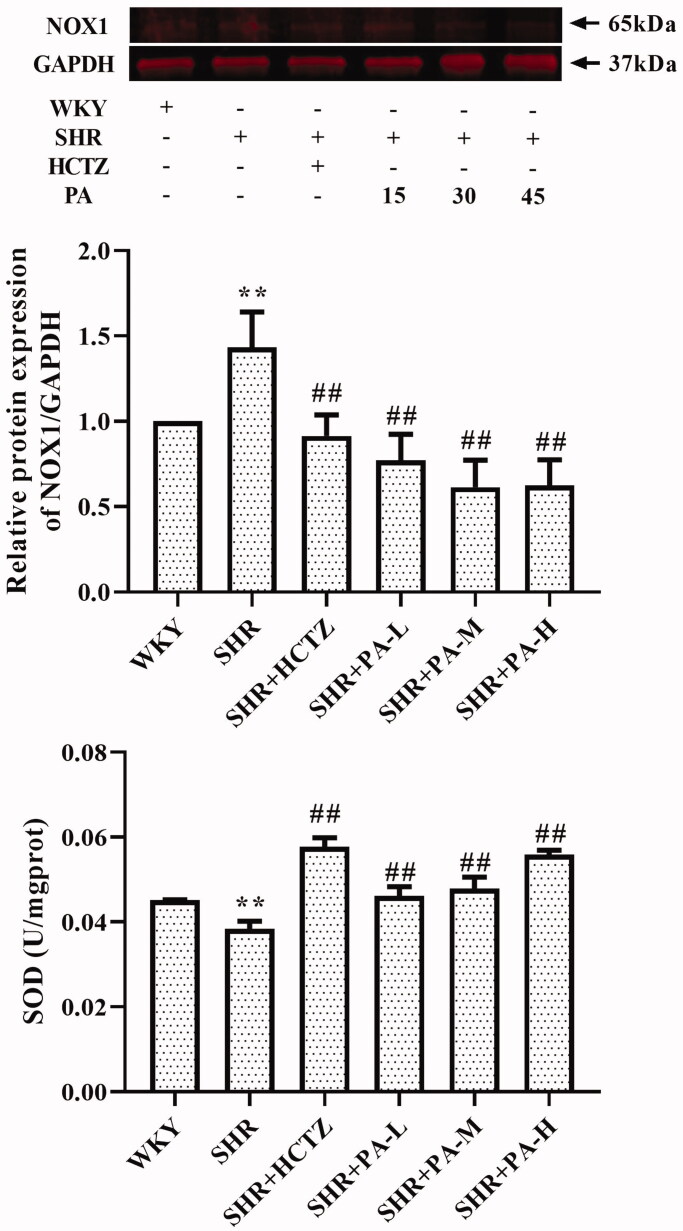
Effect of phenylacetamide on NOX1 and SOD in the hearts of SHR rats. The value of the WKY group of NOX1 was set at 1, and the relative value was presented as fold induction to that of the WKY group. Values are expressed as the mean ± SD of 6 samples. *p < 0.05, **p < 0.01 vs. WKY group; #*p* < 0.05, ##*p* < 0.01 vs. SHR group.

The method of detecting the content of SOD in serum is WST-1, and the level of NOX1 in myocardial tissue homogenate was detected by western blot and immunohistochemistry. The results showed that SOD levels in the SHR group were lower than those in the WKY group (*p* < 0.01), and NOX1 was increased (*p* < 0.01), suggesting that oxidative stress exists in SHR rats. After 3 weeks of drug intervention, it was found that all three doses of phenylacetamide increased serum SOD level (*p* < 0.01) and decreased NOX1 level (*p* < 0.01) to improve oxidative stress. Among them, the best effect is at high doses (Figures 2 and 6).

### Effect of phenylacetamide on the MAPK and TGF-β signalling pathways in SHR rats

**Figure 7.**
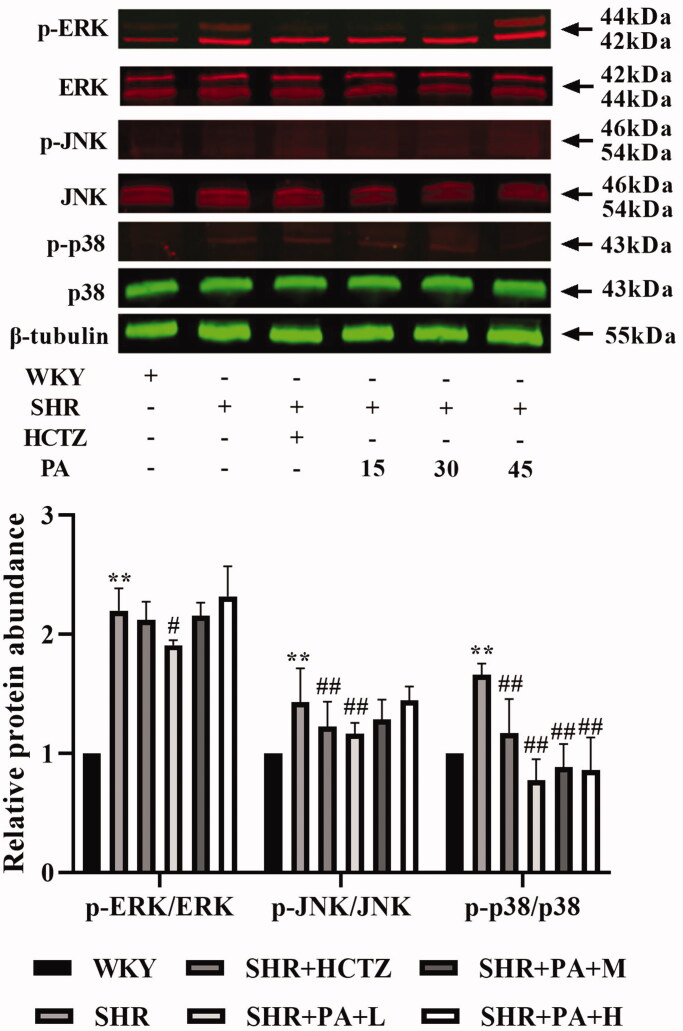
Effect of phenylacetamide on MAPK family key proteins in the hearts of SHR rats. The value of the WKY group was set at 1, and the relative value was presented as fold induction to that of the WKY group. Values are expressed as the mean ± SD of 3 samples. *p < 0.05, **p < 0.01 vs. WKY group; #*p* < 0.05, ##*p* < 0.01 vs. SHR group.

**Figure 8.**
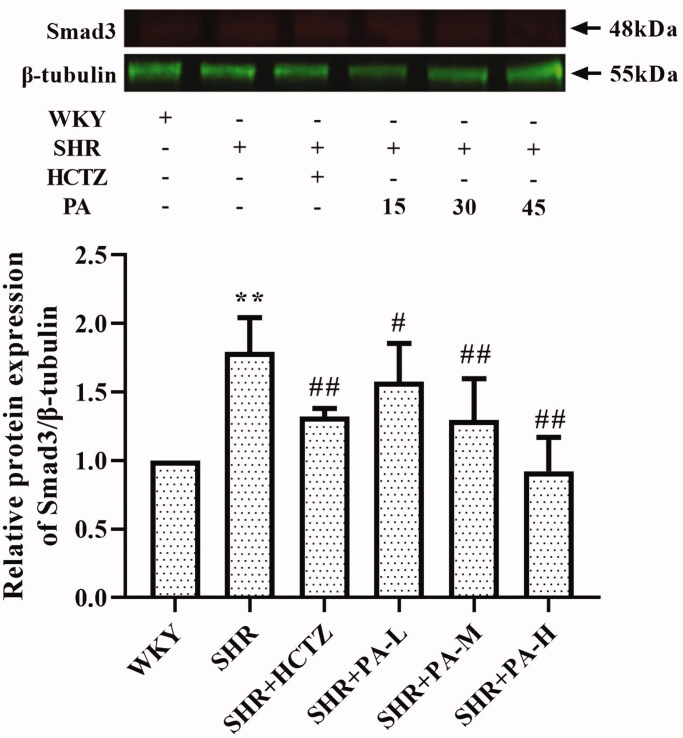
Effect of phenylacetamide on Smad3 the in hearts of SHR rats. The value of the WKY group was set at 1, and the relative value was presented as fold induction to that of the WKY group. Values are expressed as the mean ± SD of 3 samples. *p < 0.05, **p < 0.01 vs. WKY group; #*p* < 0.05, ##*p* < 0.01 vs. SHR group.

The phosphorylation levels of ERK, JNK and p38 and the expression of Smad3, the key receptor of the TGF-β superfamily, in the total protein of myocardial tissue were detected by western blot, respectively. The results showed that, compared with the WKY group, the levels of p-JNK/JNK, p-p38/p38 and Smad3 in the SHR group were significantly increased (*p* < 0.01), and the p-ERK/ERK also showed an increasing trend. Compared with the SHR group, low-dose phenylacetamide can correct p-JNK/JNK, p-ERK/ERK, p-p38/p38 and Smad3 (*p* < 0.05 or *p* < 0.01), and medium- and high-dose can correct p-p38/p38 and Smad3. Low doses have a better effect on MAPK signalling pathway, while high doses have better effects on TGF-β signalling pathway (Figures 7 and 8).

### Effect of phenylacetamide on the survival rate of H9c2 cells

**Figure 9.**
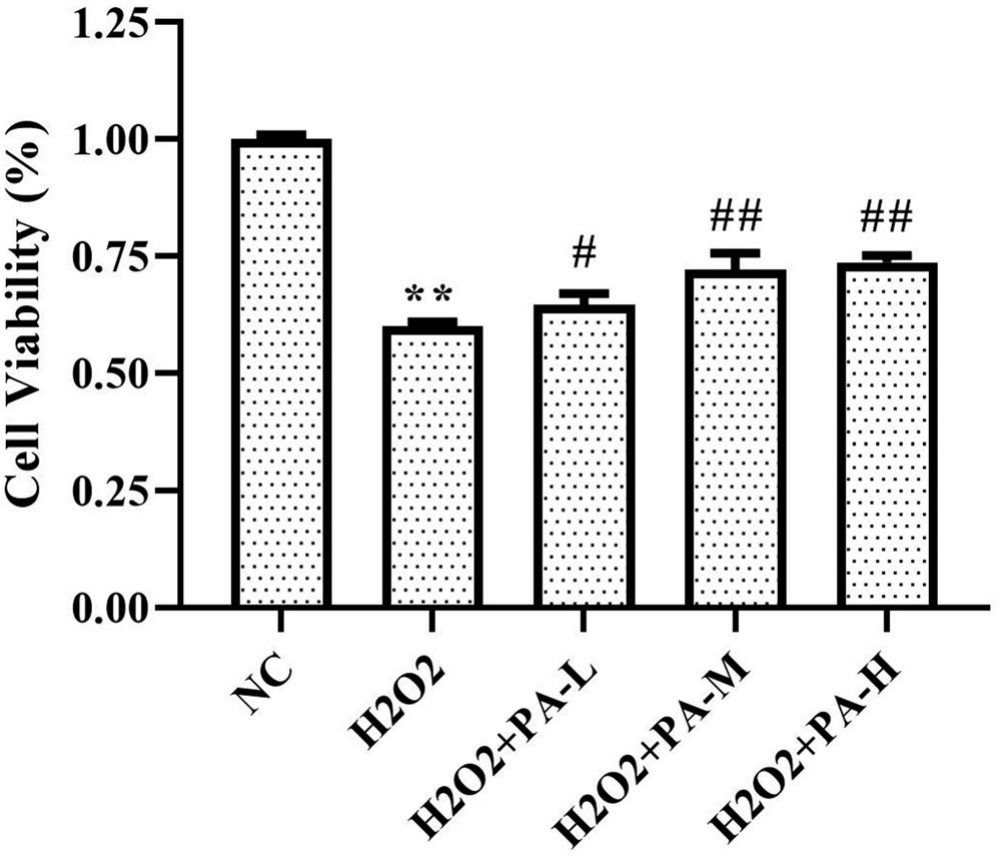
Effect of phenylacetamide on the survival rate of H9c2 cells. The value of the NC group was set at 1, and the relative value was presented as fold induction to that of the NC group. Values are expressed as the mean ± SD of 6 samples. *p < 0.05, **p < 0.01 vs. NC group; #*p* < 0.05, ##*p* < 0.01 vs. H_2_O_2_ group.

The survival rate of H9c2 cells was tested by MTT assay. The results showed that the survival rate of the H_2_O_2_ group was significantly lower than that of the NC group (*p* < 0.01). After cell protection with phenylacetamide, the cell survival rate was significantly increased (*p* < 0.01), wherein 10 μM is the optimum concentration (Figure 9).

### Effect of phenylacetamide on oxidative stress markers in H9c2 cells

**Figure 10.**
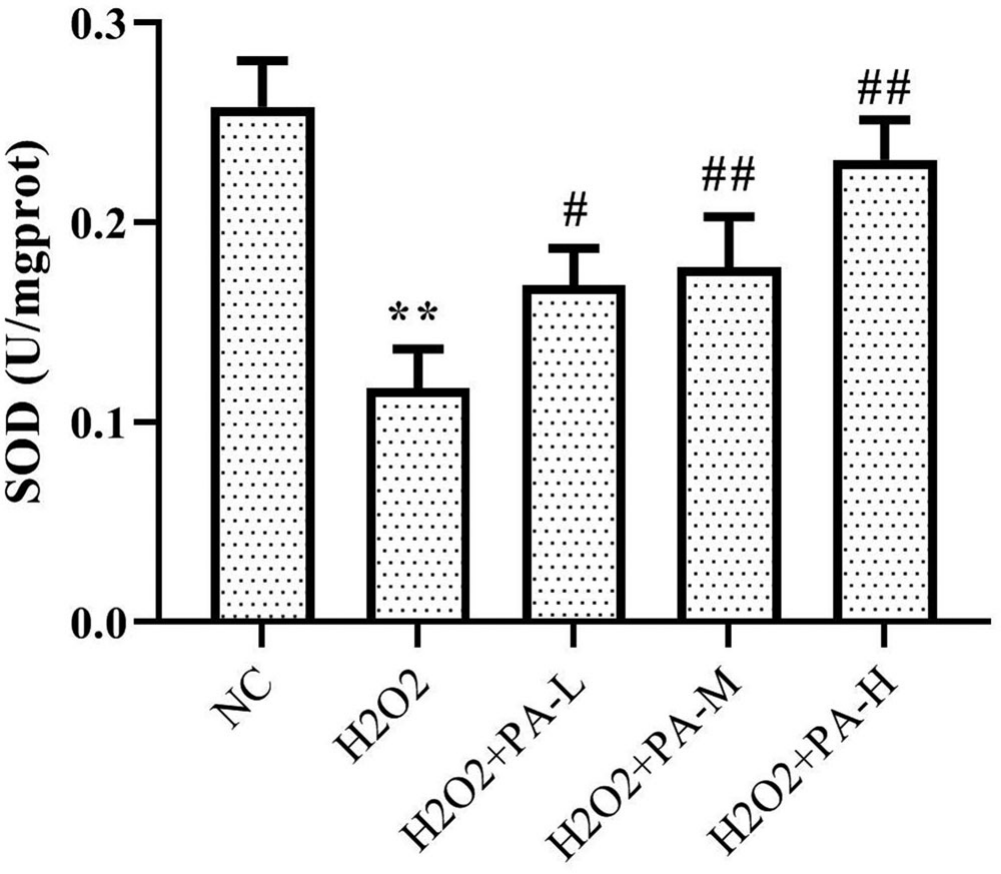
Effect of phenylacetamideon SOD levels in H9c2 cells. Values are expressed as the mean ± SD of 6 samples. *p < 0.05, **p < 0.01 vs. NC group; #*p* < 0.05, ##*p* < 0.01 vs. H_2_O_2_ group.

**Figure 11.**
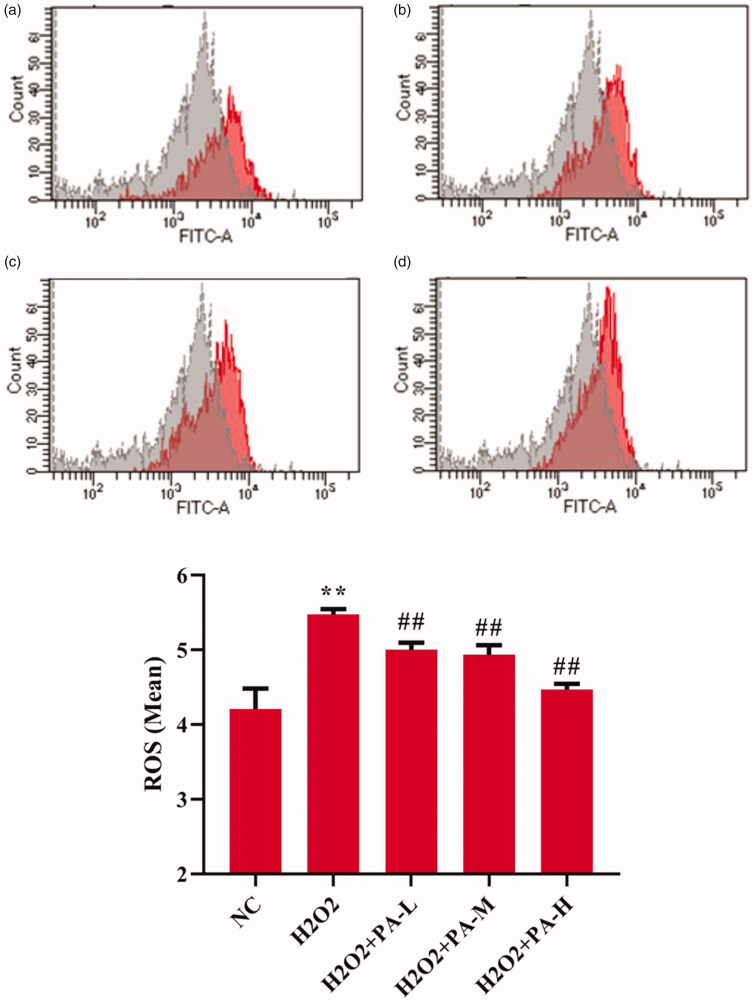
Effects of phenylacetamideon ROS levels in H9c2 cells. Figure a. b. c. d. Grey peaks indicate NC group, red peaks indicate H_2_O_2_ group, H_2_O_2_ + PA-L group, H_2_O_2_ + PA-M group and H_2_O_2_ + PA-H group, respectively. Values are expressed as the mean ± SD of 3 samples. *p < 0.05, **p < 0.01 vs. NC group; #*p* < 0.05, ##*p* < 0.01 vs. H_2_O_2_ group.

To investigate the effect of phenylacetamide on oxidative stress markers, the activity of SOD and level of ROS in H9c2 cells were tested. The results showed that compared with the NC group, the SOD activity of the H_2_O_2_ group was significantly decreased (*p* < 0.01), and the ROS level was significantly increased (*p* < 0.01). After the cells were protected with phenylacetamide, the SOD activity of the cells was significantly increased (*p* < 0.01), and ROS level were significantly reduced, wherein 10 μM is the optimum concentration (*p* < 0.01) (Figures 10 and 11).

### NOX1 and Smad3 levels

**Figure 12.**
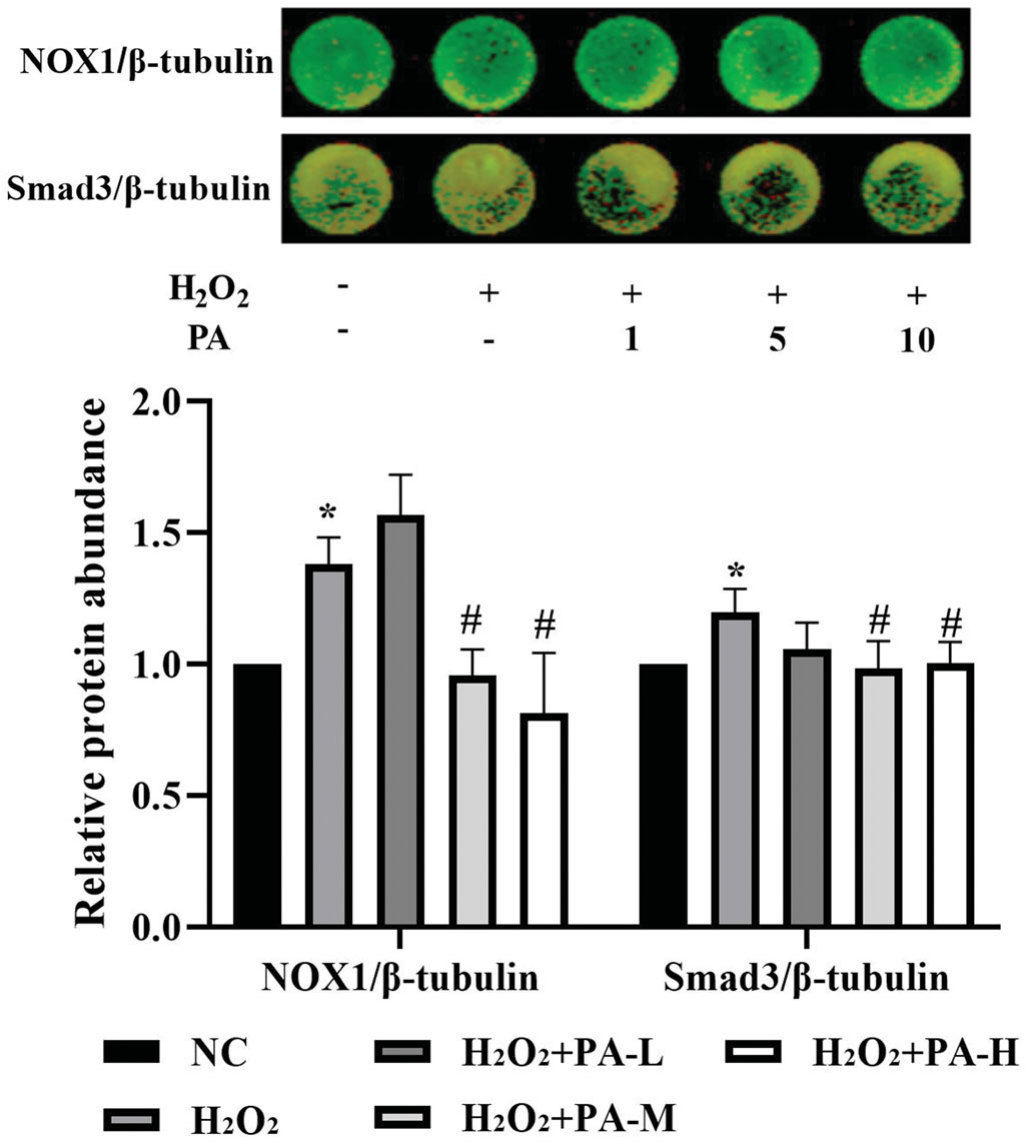
Effects of phenylacetamide on NOX1 and Smad3 in H9c2 cells. The colour in the circle represents the composite colour of the corresponding target protein (red) and β-tubulin (green). The value of the NC group was set at 1, and the relative value was presented as fold induction to that of the NC group. Values are expressed as the mean ± SD of 6 samples. *p < 0.05, **p < 0.01 vs. NC group; #*p* < 0.05, ##*p* < 0.01 vs. H_2_O_2_ group.

The NADPH oxidase NOX1 and the TGF-β signaling pathway protein Smad3 levels in H9c2 cells were examined by in-cell western. The results showed that compared with the NC group, the level of NOX1 and Smad3 protein in the H_2_O_2_ group were significantly increased (*p* < 0.05). After the cells were protected with phenylacetamide, the levels of NOX1 and Smad3 protein were significantly decreased, wherein 10 μM is the optimum concentration (*p* < 0.05) (Figure 12).

## Discussion

When peripheral intravascular pressure increases (hypertension), the mechanical load on the heart increases. To overcome this pressure overload and maintain adequate cardiac output, cardiac contractility must be increased accordingly. However, persistent overload can cause this compensatory contraction to gradually deteriorate into a decompensated contraction, destroying the heart pumping function (Rienzo et al. 2012). As shown in Figure 2, the heart tissue structure of rats in the SHR group is significantly changed, and CVF is significantly increased. Changes in the heart structure will affect the heart’s normal physiological function. But all three doses of phenylacetamide can improve the pathological structure of rat heart and reduce CVF.

Echocardiography is a common method for detecting heart function. Left ventricular pumping is the result of a combination of left ventricular preload, afterload and myocardial contractility, which can be significantly affected by left ventricular pre and posterior loading. Therefore, in the detection of echocardiography, to correct the effect of left ventricular anterior and posterior loading, it is necessary to further calculate LVEF and LVFS on the basis of detecting LVEDD and LVESD. As shown in Figure 3, in the SHR group, LVEF and LVFS decreased, suggesting that the left ventricular pumping function of SHR rats was reduced, but medium- and high-dose phenylacetamide can significantly improve heart function. High dose works best.

When the myocardium is damaged, the BNP gene in cardiomyocytes is activated and rapidly expressed to produce BNP and NT-proBNP (Stämpfli et al. 2017). Although BNP and NT-proBNP are always secreted in equimolar amounts, the former has a slow clearance rate, a long half-life (Bajoria et al. 2003) and high stability. Therefore, NT-proBNP is receiving increasing attention from researchers in clinical testing. Studies have shown a significant negative correlation between NT-proBNP and left ventricular ejection fraction (Fett et al. 2004). At the same time, myocardial damage can also make the serum CKMB level raises within 3-8 h, reach a peak within 9-30 h and return to normal within 48-72 h. Because it is mainly present in cardiomyocytes and has strong specificity, it is currently one of the reliable indicators for early diagnosis of myocardial damage (Kavsak et al. 2007). As shown in Figure 3, the levels of NT-proBNP and CKMB in the SHR group were significantly increased, further verifying the myocardial injury in SHR. Phenylacetamide improves both, but low- and high-doses have different treatment tendencies. The best improvement for CKMB is the low dose, but the best improvement for NT-proBNP is the high dose, which suggests that the molecular mechanism of phenylacetamide may be different under different dose conditions.

The RAS system is an important regulatory system in the development of hypertension and various cardiovascular diseases. In particular, excessive activation of local RAS located in the heart plays an important role in the progression of myocardial injury caused by high blood pressure (Mendelsohn and Karas 2005). *In vitro* cultured cardiomyocytes and fibroblasts contain a variety of RAS components (Hollenberg 1999), and there are also receptors for these components in the myocardial tissue (Collins 2002). Numerous studies such as these have confirmed that local RAS is an independent system. The effects of phenylacetamide on REN and ACE in myocardial homogenate were investigated. As shown in Figure 4, the results showed that the level of REN in myocardial tissue of SHR was significantly higher than that of normal rats, and the level of ACE also has a certain upward trend. After treatment with phenylacetamide, the levels of the two substances were significantly reduced. Among them, low doses have the best therapeutic effect, which indicates that phenylacetamide improves myocardial damage by inhibiting the RAAS system at low doses.

Persistent hypertension can lead to endothelial damage and ET-1 secretion increases when endothelial cells are destroyed. ET-1 aggravates the condition of hypertension through its own vasoconstriction effect. It can exert chemotaxis on neutrophils and monocytes, causing them to accumulate in the injury site and stimulate them to secrete a large number of inflammatory factors, such as interleukin-1β (IL-1β) etc. (Fang et al. 2019). IL-1β can stimulate the healing of the affected area by promoting the secretion of inflammatory proteins by cells, such as monocytes, macrophages or endothelial cells (Kashyap et al. 2017). It is destructive to the myocardium and can be involved in myocardial damage caused by hypertension by mediating ventricular remodelling and promoting cardiomyocyte apoptosis (Mahmoudi et al. 2016). The expression of IL-1β is upregulated in ventricular hypertrophic animals (Bujak and Frangogiannis 2009). At the same time, in experimental hypertensive animals, the severity of hypertension is positively correlated with the level of ET-1 (Desideri et al. 1997). This is consistent with our experimental results. As shown in Figure 5, compared with the WKY group, the levels of ET-1 and IL-1β in the myocardial tissue homogenate of the SHR group were significantly increased, but after 3 weeks of treatment with phenylacetamide, levels of both inflammatory factors were significantly improved. Among them, the low-dose effect is the best, indicating that phenylacetamide improves myocardial damage by regulating inflammatory factors at low doses.

Persistent hypertension induces mitochondrial adenosine triphosphate (ATP)-dependent potassium channel opening, stimulates mitochondrial synthesis and secretes more ROS, triggering oxidative stress (De Giusti et al. 2013). Oxidative stress is an imbalance between ROS levels and insufficient antioxidants in the body. Insufficient antioxidants lead to insufficient ROS scavenging capacity, which eventually leads to accumulation of ROS and causes intracellular oxidative damage (Lassegue and Clempus 2003). SOD is an important antioxidant enzyme and the primary substance for scavenging free radicals in the body (Mendelsohn and Karas 2005). There is an ROS rapid production enzyme, NADPH oxidase (NOX), which is a multi-subunit complex. NOX1 is a homolog of NOX, present in the cytoplasmic membrane microcapsule (Paik et al. 2011), and they have similar structures and functions (Lippai et al. 2013). When oxidative stress occurs in the body, the subunits of NADPH oxidase and their homologs will also change accordingly. As shown in Figures 2 and 6, compared with the WKY group, the SOD activity of the SHR group decreased, and the NOX1 protein expression increased, suggesting the appearance of oxidative stress. However, after treatment with phenylacetamide, this situation has improved, and unlike before, this time is the best effect with high doses. Considering that high doses have better effects on improving most of the markers of myocardial injury in SHR rats, we speculate that the improvement of phenylacetamide on hypertension and cardiac function may depend more on its antioxidant capacity.

The MAPK signalling pathway is over-activated by the induction of various inflammatory cytokines (Feng et al. 2017). The MAPK family ERK1/2, JNK and p38 MAPK are the intracellular signal transduction protein kinases that are most closely related to cell proliferation and hypertrophy, and their activation plays an important role in promoting myocardial growth and development. The study finds that the MAPK signalling pathway is over-activated in pathological ventricular hypertrophy (Rose et al. 2010). In SHR, the expression of p38 MAPK in cardiomyocytes is continuously elevated during cardiac hypertrophy (Behr et al. 2001). ERK1/2 and JNK can also coordinate with each other to regulate cardiomyocyte hypertrophy (Sugden 1999). As shown in Figure 7, the results of this experiment showed that the ERK1/2, JNK, and p38 phosphorylation levels were significantly increased in the SHR group compared with the WKY group, but the levels of the three proteins were significantly reduced after phenylacetamide treatment. Among them, the low-dose treatment is the best, so we speculate that the improvement of low-dose phenylacetamide on myocardial injury may be related to the MAPK signalling pathway.

To further explore the effects of phenylacetamide on hypertension and cardiac function, we examined the expression of Smad3 protein. SMAD is an important protein that mediates the development of various cardiovascular diseases. In SHR, myocardial hypertrophy and the total quantity and properties of collagen fibres can be improved by affecting the level of Smad3 (Yuan and Jing 2010). Some scholars have confirmed that Smad3 overexpression induces oxidative stress, while inhibition of its expression reduces oxidative stress (Yuan and Jing 2010). As shown in Figure 8, the results showed that Smad3 level was significantly elevated in the SHR group compared with the WKY group, but Smad3 level was significantly reduced after 3 weeks of phenylacetamide treatment. And the improvement trend is consistent with its antioxidant effect, which is the best treatment for high doses. Therefore, we speculate that high-dose phenylacetamide may improve oxidative stress by regulating the expression of Smad3 protein, thereby improving myocardial damage in hypertensive rats.

According to the above experiments, we found that low doses of phenylacetamide inhibit the inflammation and RAAS system by regulating the MAPK signalling pathway, thereby improving myocardial damage. However, with the increase of dose, its mechanism of action gradually changed. High doses of phenylacetamide exert antihypertensive and cardioprotective effects by regulating Smad3 protein expression and oxidative stress. However, we are still not sure whether phenylacetamide is indirectly preventing myocardial damage by lowering blood pressure, or is it a direct cardioprotective effect. So, we used H_2_O_2_ to stimulate H9c2 cells to mimic direct myocardial damage caused by oxidative stress. As shown in Figures 9–12, the model group cells were found to have oxidative stress, and the expression of Smad3 protein was increased, which eventually led to a decrease in cell survival rate. However, phenylacetamide can still improve oxidative stress, reduce Smad3 protein expression, and increase cell survival rate. This confirms that the cardioprotective effect of phenylacetamide at high doses is a direct effect to some extent.

## Conclusions

**Figure 13.**
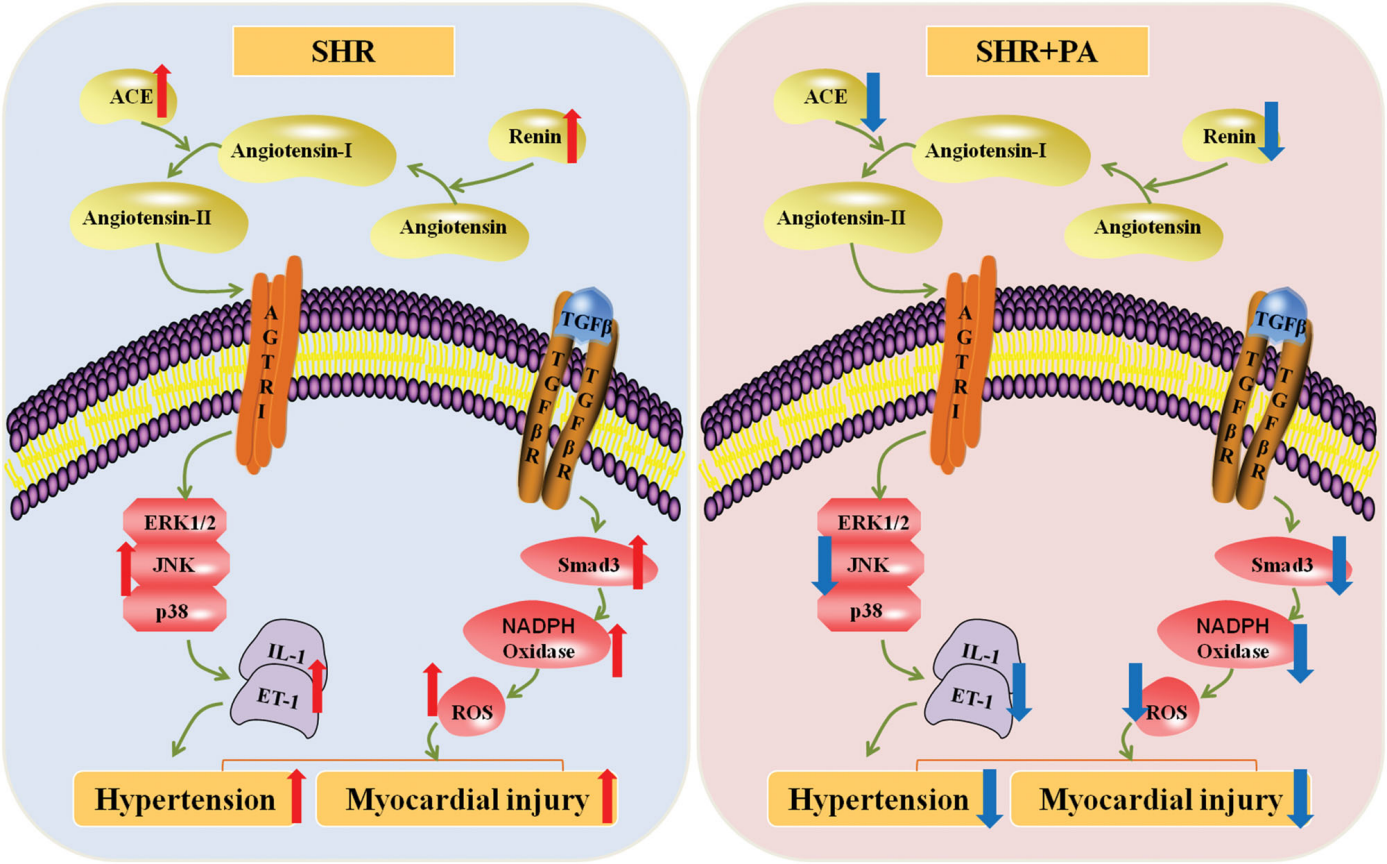
Mechanism of phenylacetamide on myocardial injury in SHR. The picture on the blue background indicates the pathological manifestation of the SHR, and the red arrow indicates the pathological trend of each indicator. The picture on the pink background indicates changes after phenylacetamide treatment, and the dark blue arrow indicates the trend of improvement in each indicator.

As shown in Figure 13, phenylacetamide can improve myocardial damage by inhibiting MAPK signalling pathway, inflammation and RAAS system at low dose of 15 mg/mL. With increasing dose, phenylacetamide improved myocardial damage in hypertensive rats at dose up to 45 mg/mL, mainly by reducing Smad3 protein expression and inhibiting oxidative stress.
